# Republication of “FAO: The Open Source for High-quality Peer Reviewed Orthopaedic Foot and Ankle Content”

**DOI:** 10.1177/24730114231188081

**Published:** 2023-07-17

**Authors:** L. Daniel Latt

**Commentary:** This editorial introduces the reader to the new journal *Foot & Ankle Orthopaedics* and dispels common myths about open access journals.

Welcome to *Foot & Ankle Orthopaedics(FAO)*, the new open access scholarly journal of the American Orthopaedic Foot & Ankle Society (AOFAS). This medical journal covers the surgical and medical management of foot and ankle disorders with a specific focus on reconstructive, trauma, and sports-related conditions utilizing the latest technological advances.

For those who may not be familiar with open access journals (OAJ), I would like to briefly explain the concept (an excellent overview of OAJ can be found here^5^) and dispel some myths. An OAJ is a scholarly journal that is available online to the reader “without financial, legal, or technical barriers other than those inseparable from gaining access to the internet itself.”^1,4^ Open access publication began with the formation of the internet in the early 1990s, but was not formalized until a series of meetings (most notably the Budapest Open Access Initiative) occurred in the early 2000s.^2^ The popularity of OAJs increased slowly at first, but has experienced exponential growth in the past few years. The Directory of Open Access Journals now listing nearly 10,000 journals.^3^ Many federal funding programs currently require open publication of works resulting from their funding and many academic institutions cover the cost of publication for their faculty in open access journals.

There are 2 common myths about open access journals. The first is that open access journals are not peer reviewed or have lesser editorial standards compared to traditional journals. The truth is that the editorial policies and standards of a journal are independent of its financial model. The second myth is that authors pay for the privilege of getting their article published. The unfortunate reality is that there are costs associated with publishing a journal and these costs must be covered somehow. In a traditional print journal, the cost of publication is covered through subscription and advertising fees, whereas in an open journal, these costs are covered by the article-processing charge (APC). In essence, the authors pay a fee so that their readers do not have to. Open access allows the information to reach readers who lack an individual or institutional subscription—an all too common barrier for trainees and for practicing physicians in the developing world.

*FAO* was created as a sister journal to *Foot & Ankle International* (*FAI*) to meet the needs of AOFAS members, and other orthopedic surgeons, fellows, and residents, for an additional outlet for their scholarly work and as a way to share information worldwide. *FAO* adheres to the same rigorous peer review standards as its sister publication. *FAI* and *FAO* will, at the start, share the same pool of reviewers.

OAJs are not new in orthopaedics. We are fortunate to be able to follow the model established by the American Orthopaedic Society for Sports Medicine (AOSSM) with the launch of the *Orthopaedic Journal of Sports Medicine (OJSM)* as a sister publication to the *American Journal of Sports Medicine* 3 years ago. The rapid growth of *OJSM* demonstrates that a subspecialty society can successfully support both traditional print and open online journals.

*FAO* will be published online and will be freely available worldwide via the internet. The online-only publication has a number of advantages, including: more rapid publication, lower cost, and flexibility in content and format. The journal will feature a diverse mix of content, including: original clinical and basic science research, narrative reviews, systematic reviews, metaanalysis, case reports, hot topics, research designs, and technique articles. Additionally, *FAO* will contain a number of format enhancements, including videos, hypertext links, and scripting, which will make for a dynamic reader experience. Although *FAO* (like other new journals) is not currently indexed in Medline, it is our goal to become so as soon as possible, and once indexed, all previously published articles will be retroactively included.

The *FAO* editorial board, assistant editors, and I are excited to open this new chapter in orthopaedic foot and ankle research. We hope to make this journal the ideal resource for orthopaedic surgeons, fellows, residents, and allied health providers worldwide who seek the latest in evidence-based foot and ankle information. We hope that you will return to this website frequently to see what is new in foot and ankle surgery, to download and share articles, and to contribute your work to this new repository of orthopaedic foot and ankle knowledge that is freely available worldwide.


L. Daniel Latt, MD, PhD *Editor-in-Chief*

